# Advancements in mapping areas suitable for wetland habitats across the conterminous United States

**DOI:** 10.1016/j.scitotenv.2024.175058

**Published:** 2024-07-29

**Authors:** Lauren Krohmer, Elijah Heetderks, Jeremy Baynes, Anne Neale

**Affiliations:** a Oak Ridge Associated Universities supporting U.S. Environmental Protection Agency (EPA), Center for Public Health and Environmental Assessment (CPHEA), 109 T. W. Alexander Drive, Research Triangle Park, NC 27711, USA; b U.S. Environmental Protection Agency (EPA), Center for Public Health and Environmental Assessment (CPHEA), Environmental Pathways Modeling Branch (EPMB), 109 T.W. Alexander Drive, Research Triangle Park, NC 27711, USA

**Keywords:** Machine learning, Predictive modeling, Training data, Ecosystem services (and/or nature-based solutions), Restoration (and/or conservation)

## Abstract

Wetland habitats provide critical ecosystem services to the surrounding landscape, including nutrient and pollutant retention, flood mitigation, and carbon storage. Wetland connectivity to water bodies and related ecosystems is critical in habitat sustainability, but there are limited resources for landscape-level wetland planning. Considering the network connectivity of an ecosystem type can derive different benefits to the natural and built environment, as well as human health. The value that wetlands provide, along with incentive programs and conservation goals mandated by the government require new and improved wetland spatial data. Utilizing high quality, publicly available data, this study finds that the amount of land in the United States that could support built or restored wetlands is more than double the area of mapped existing wetlands.

This study uses 17 input variables (i.e., features extracted from remotely sensed data and auxiliary datasets) at the 10-m resolution and the National Wetlands Inventory to train a random forest model to identify areas that may support a wetland habitat, or potential wetland areas. Models were calculated for each of 18 two-digit hydrologic units that encompass the conterminous United States, and model overall accuracy ranged from 78.0 % to 89.8 %. The models predicted that 21.1 % of the conterminous United States can be categorized as potential wetland area.

Selecting input variables to predict areas with wetland potential, rather than to identify existing wetlands, using the random forest algorithm can be transferred to other locations, scales, and ecosystem types. Visualizing potential wetland areas using input data at the 10-m resolution and enhanced methodology improves previous work, as even slight changes in topography, soils, and landscape features can determine ecosystem connections. This product can be used to better place wetland restoration projects to serve ecosystem- and community-wide health by ensuring ecosystem success and targeting areas that face increased climate change impacts.

## GRAPHICAL ABSTRACT

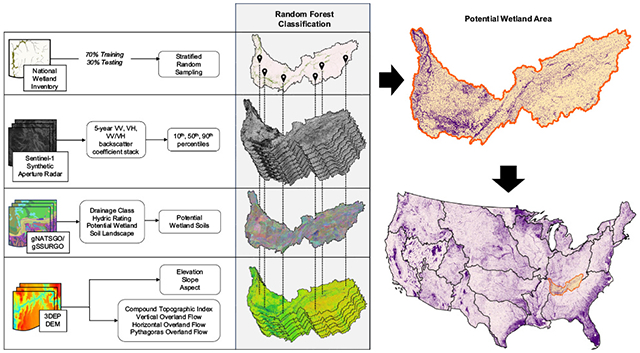

## 1. Introduction

It is well known that wetlands provide a wide range of ecosystem services including carbon storage, flood mitigation, sediment and nutrient retention, wildlife habitat, and access to recreational activities (Adusumilli, 2015; Horvath et al., 2017). Researchers have estimated that up to 53 % of wetlands in the conterminous United States (CONUS) have been lost since the 1600s, and remaining wetland habitats face continued threats of loss or degradation from human activities (O’Neil et al., 2018; Tang et al., 2012). The National Wetlands Inventory (NWI) is the only publicly available resource that maps wetland habitats across the United States and its territories (United States Fish and Wildlife Service, 2021). The NWI is developed from aerial imagery and field assessments, some of which dates to the 1970’s, with targeted water body feature inclusion from the U.S. Geological Survey’s National Hydrography Dataset (NHD) (U.S. Geological Survey, 2022). Given the importance of wetlands, historic high rate of loss, continued threats from land-use change, and other challenges facing ecosystem health (e.g., climate change and related disasters), up-to-date and high-quality resources that can support these valuable habitats will continue to be sought after.

Section 404 of the Clean Water Act protects wetlands and other aquatic resources from certain activities, and requires compensatory mitigation for impacts incurred (Adusumilli, 2015). Voluntary conservation programs, like the Wetland Reserve Enhancement Partnership (WREP) administrated by the Natural Resources Conservation Service (NRCS) and the Farmable Wetland Program (FWP) administered by the Farm Services Agency (FSA) which are both under the U.S. Department of Agriculture (USDA), encourage farmers to restore wetlands with incentives such as rental payments (Farm Services Agency; NRCS, 2023). Between 2001 and 2016, croplands accounted for 58 % of all land in the United States converted to wetland (Taylor and Druckenmiller, 2022). A meta-analysis by the U.S. EPA and U.S. Army Corps of Engineers estimates the average annual value of ecosystem services produced by wetlands protected by the Clean Water Act to be between $39,000 and $76,000 (2020 USD) per hectare (U.S. Environmental Protection Agency, 2021). Additionally, the Millennium Ecosystem Assessment found that intact freshwater marshes have an economic value of approximately $7700 per hectare compared to $3200 (2020 USD) per hectare when converted for agriculture (Millennium Ecosystem Assessment, 2005). The monetary support for restoration efforts, and estimated value of these ecosystems, further highlight a need for increased spatial data concerning wetlands, and the landscape characteristics that promote ecosystem growth.

Wetland restoration efforts typically do not consider the larger landscape effects of wetland location or function, even though the success of restored and built wetlands rely on landscape-scale factors such as topography, soils, and hydrology (Caldwell et al., 2011; Cheng et al., 2020; Horvath et al., 2017; Tang et al., 2012). Connectivity to nearby water and wetland habitats supports successful ecosystem functioning and should be considered alongside other landscape features to determine wetland restoration sites (Babbar-Sebens et al., 2013; Leibowitz et al., 2023; White and Fennessy, 2005). Considering wetland restoration at the regional, state, or watershed scale can provide targeted ecosystem services, including nutrient removal and improved water quality. There currently exists a gap in knowledge to support best placement of restored or built wetlands, which would improve connectivity and ecosystem services provided by these systems (Cheng et al., 2020). This study seeks to use data about existing wetlands to predict areas across the conterminous United States that are likely to support wetland habitats, or potential wetland area (PWA).

The random forest (RF) algorithm is frequently used in land cover classification, with more accurate results compared to other prediction models (Forkuor et al., 2017; Phan et al., 2020). Many recent studies make use of machine learning (ML) with large datasets, that are both larger in spatial range as well as resolution, to support land cover modeling (Gumma et al., 2020; Mahdianpari et al., 2020a; Tamiminia et al., 2020; Teluguntla et al., 2018). Large datasets like these can become unwieldy with data storage and cost, but technologies like Google Earth Engine (GEE) make large datasets more manageable (Gorelick et al., 2017; Phan et al., 2020). The application of cloud computing in GEE allows for faster processing of large datasets, making a 10-m resolution product for CONUS possible with reduced local computing and memory needs.

Land cover classification utilizing the RF algorithm and remotely sensed imagery are improved with the addition of auxiliary data, such as topographic and landscape variables (Corcoran et al., 2013; Zeferino et al., 2020). This study follows a relatively smaller set of research that predicts areas that would likely support a wetland ecosystem, rather than identifying land cover classes that are currently present on the ground (Crouzeilles et al., 2020). This difference in research goal influenced the selection of training data (i.e., existing wetlands) and predictive landscape factors to identify areas with a high likelihood of supporting a wetland habitat. This paper builds on previous methodologies, laid out in Horvath, et al. (Horvath et al., 2017), which utilized the Compound Topographic Index (CTI) and soil drainage characteristics to identify locations where built or restored wetlands could thrive across the United States. Our study seeks to expand upon this work by increasing the resolution from 30-m to 10-m, incorporating ML, and utilizing additional variables to identify PWA across CONUS.

The primary final product of this study is a gridded dataset with 10-m resolution that identifies areas where the landscape, land use, and hydrology would likely support a wetland habitat. We also overlaid those data with areas of agricultural land use to identify areas that may be suitable for voluntary conservation programs to identify PWA on existing croplands. These products are intended to provide a guide for practitioners and policymakers that highlight areas likely to support wetland ecosystems, which can be used in tandem with spatial data about existing wetlands to improve restoration and conservation outcomes.

## 2. Methods

We used the level-2 hydrologic unit code (HUC-2), which divides CONUS into 18 watersheds, as our model area of interest (Table 1). The average size of a HUC-2 region is approximately 438,700 km^2^ (U.S. Geological Survey, 2013). This scale was chosen to capture regional climatic and physiographic effects on wetland presence within the training data and consider the processing limits of Google Earth Engine (Collins et al., 2022; Woznicki et al., 2019). Due to constraints in data availability, HUC-2 s were clipped where they extended past the United States boundary into Mexico or Canada. Outlying U.S. states and territories have inconsistent data coverage for some input variables and were not considered in this study. The term “variable” is used to refer to features extracted from remotely sensed data, as well as auxiliary geographic data.

### 2.1. National Wetlands Inventory

The NWI maps existing wetlands for the United States utilizing historic and recent aerial photography and field assessments to identify and classify wetlands (Horvath et al., 2017; Wilen and Bates, 1995). The NWI classifies wetlands into eight broad systems which are further classified by subsystem, class, and subclass (Cowardin et al., 1979). The NWI has been used in other studies as reference data to ensure correct identification of training data or to compare wetland classification following a ML method (Muñoz et al., 2019; O’Neil et al., 2018). The inventory is updated twice per year, and the state products released in October 2021 were used in this study (United States Fish and Wildlife Service, 2021). While the NWI is the most consistent product available at the CONUS scale, it is important to note that some parts of the country are based on imagery from as early as the 1970s, and there are some areas that have not been mapped. All NWI wetlands were considered, despite the difference in imagery dates, because these sites are likely to have some characteristics of a wetland ecosystem maintained, even if the areas have since been converted to another use.

The NWI was used to designate our wetland and non-wetland classes for the training and validation pixels used in the modeling process. Considering pixels individually, rather than using known wetlands as objects, enhances the ability for the model to highlight small areas that may support wetland habitat. Additionally, pixel-based approaches have been shown to yield similar accuracy to object-based studies when looking at wetlands on agricultural land types (Corcoran et al., 2015). State-level NWI feature layers were merged into a CONUS-wide layer, and wetland subsystems were designated as part of the “wetland” class, “non-wetland” class, or were not included in our dataset (Table 2).

All marine systems and subtidal estuarine subsystems were considered non-wetland for this study, which ensures that the modeling algorithm was not trained to identify wetlands on permanent deep-water and open-ocean systems. The reclassified NWI was then converted to a binary raster, where the “wetland” class was assigned a value of “1”, classes not included were assigned ‘NoData’, and any area outside the extent of the selected “wetland” class was considered “non-wetland” and given a value of “0”. The resulting binary raster is referred to as the ‘wetlands binary raster’ for the remainder of this paper.

### 2.2. Topographic variables

To identify potential wetlands across CONUS, datasets with full U.S. coverage were used (Table 3). Nationwide digital elevation model (DEM) data were sourced from the U.S. Geological Survey (USGS) at 1/3 arc-second resolution from the agency’s 3D Elevation Program (3DEP) (Stoker and Miller, 2022; U.S. Geological Survey, 2020). This product has an overall vertical accuracy of 0.82 m (root mean square error), with variance across the dataset (Stoker and Miller, 2022). Missing data (‘NoData’) in DEM tiles were interpolated using the GDAL fillnodata Python script with a max distance of 15 pixels (GDAL/OGR contributors, 2022). DEM tiles were mosaicked, projected to Albers Equal Area Projection at 10-m resolution, and clipped to a modified version of the U.S. Census Bureau’s, 2021 U.S. state boundaries to include all available DEM data (U.S. Census Bureau, 2021). The modified DEM data were used to calculate the CTI, the vertical component of the overland flow distance to channel (VOFD), the horizontal component of the overland flow distance to channel (HOFD) to channel, and the Pythagoras (i.e., straight line) component of the overland flow distance to channel (POFD) using Terrain Analysis Using Digital Elevation Models (TauDEM 5.3.7) Toolbox (Tarboton, 2005). These flow distance measures represent hydrologic connectivity on the landscape which is important in wetland ecosystems that rely on hydric conditions (Dubeau et al., 2017).

TauDEM provides two common methods for modeling flow direction and accumulation from a DEM. The D-8 method models flow from each pixel into one of the neighboring eight pixels while the D-infinity method proportions flow between the cells with the steepest downward slope of a triangular facet (Tarboton, 1997). D-infinity is a more computationally complex calculation that has been shown to work equally as well as D-8 in most areas and has been shown to have better agreement with reference data specifically when mapping wetlands (Rampi et al., 2014). The computational requirements to apply the D-infinity method necessitated modeling a smaller area than HUC-2. Therefore, we modeled the flow direction or flow accumulation derivates (CTI, VOFD, HOFD, POFD) at the level-4 hydrologic unit code (HUC-4). Within CONUS, there are 222 HUC-4 s that nest within the 18 HUC-2 s. HUC-4 s have an average area of 40,699 km2 (σ = 22,938 km^2^).

The CONUS DEM mosaic was clipped by HUC-4 with a buffer of 6675-m added to reduce edge effects produced when calculating flow accumulation. The distance used approximately matches the buffer distance USGS provides on similar products released under their NHDPlus High Resolution value-added products (U.S. Geological Survey, 2022). For each HUC-4 DEM plus buffer area, pits were removed, and D-infinity flow direction and flow accumulation were calculated without edge contamination. A channel network raster was calculated using a flow accumulation threshold method laid out in Woznicki et al. (2019). The channel threshold defined by the Elevation Derivatives for National Applications (EDNA) from USGS was adjusted to 10-m resolution, and set such that any pixel that accumulates 45,000 pixels (4.5 km^2^) of flow is considered a channel (Johnston et al., 2009). A single-cell boundary along coastlines was added to act as the channel at coastal boundaries to represent flow into oceans and estuaries. The D-Infinity Distance Down tool was applied to the derived channel raster and the elevation raster that was created using the D-Infinity Flow Direction and Pit Remove tools to calculate HOFD, VOFD, and POFD (Tarboton, 2005). Model output for each flow distance types were spatially clipped to the original (i.e., non-buffered) HUC-4 boundary and were mosaicked to produce a 10-m CONUS data layer.

CTI is frequently used in machine learning studies applied to wetland ecosystems and has been shown to support increased accuracy in identifying wetlands when used with other inputs such as radar imagery and vegetation indices (Dubeau et al., 2017). CTI highlights areas where water is likely to accumulate on the surface (Beven and Kirkby, 1978). For our calculation of CTI, we added 0.0001 rad to the slope to prevent NoData that arise when dividing by 0, as suggested by Rampi et al. (2014). A CTI raster was calculated for each HUC-4 plus buffer area. CTI model outputs were smoothed with a 3 × 3 focal mean moving window and spatially clipped to the original (i.e., non-buffered) HUC-4 boundary. The HUC-4 CTI products were mosaicked to produce a 10-m CONUS data layer. CONUS CTI values were linearly scaled from 0 to 1000 and converted to integer data type to reduce data file sizes. Linear scaling maintained the original distribution of CTI values, and 1000 bins was an adequate precision for this effort.

Within GEE, the 3DEP elevation product was used as the elevation source and to calculate aspect and slope using GEE’s Terrain toolset (Gorelick et al., 2017). Aspect and slope are calculated using four neighboring cells and are given in degrees.

### 2.3. Soil variable

Conterminous soil data were built from the gridded National Soil Survey Geographic Database (gNATSGO) and the gridded Soil Survey Geographic Database (gSSURGO) produced by the Natural Resource Conservation Service (Soil Survey Staff, 2021a, 2021b). gNATSGO combines data from gSSURGO with two other soil survey datasets – the Raster Soil Survey (RSS) and the Digital General Soil Map of the United States (STATSGO2). At the time of this study, 26 states and the District of Columbia were processed for gNATSGO. The remaining states were filled in using gSSURGO data. All states available for both products were downloaded and mosaicked to create a seamless 10-m CONUS product.

Soil characteristics pertaining to hydrologic conductivity and drainage are useful in identifying wetland potential (White and Fennessy, 2005). The U.S. Fish and Wildlife Service, who produce the NWI used in this study, define wetlands from Cowardin et al. (Cowardin et al., 1979) as meeting at least one of three components – 1) hydrophytic vegetation periodically appears on the landscape, 2) substrate is primarily hydric soil, and 3) the substrate is non-soil and is either saturated or inundated by water at some time during the growing season every year. Because this study is identifying potential wetlands by their biophysical characteristics, the latter two of the three major components were the focus of soils data collection. The 10-m state-level gSSURGO and gNATSGO products from October 2021 were used to develop our soil input variable. (Soil Survey Staff, 2021a, 2021b).

The gSSURGO and gNATSGO products contain a Component table which includes a variety of characteristics about each component that is within a mapping unit. There are 314,135 mapping units that vary in shape and size that represent areas of similar soil characteristics. Soil components are presented as a percentage of coverage by mapping unit. The Component tables for each state were merged to create a CONUS table. For this study, we prioritized drainage class, over hydric class, but utilized both categories to reduce NoData areas. Drainage class describes the ability for soil to hold water, while the hydric rating refers to the presence of anaerobic conditions, caused by flooding or saturation. Thus, hydric soils are likely to already support wetland ecosystems, whereas drainage class can identify areas that could support a wetland, even if anaerobic conditions have not yet been attained. Drainage classes “poorly drained” and “very poorly drained” and hydric class “yes” were determined conducive to supporting PWA. Drainage class and hydric class percentages were summed per mapping unit, resulting in a percentage of hydric soil cover. gSSURGO products also contain the soil categorization “potential wetland soil landscapes” (PWSL), which provides a percentage of mapping unit covered by soil components that support wetlands (Soil Survey Staff, 2021b). This category was used to fill in null values from the drainage class and hydric class merge. The percentage that each component covers the mapping unit was summed by the mapping unit key, a unique identifier for each map unit. The result of this procedure is a percentage cover of each mapping unit by soils that are likely hydric and are referred to as Potential Wetland Soils (PWS) in this study.

### 2.4. Synthetic aperture radar variables

Sentinel-1 images for CONUS were sourced from and manipulated within GEE, which provides pre-processed scenes. The pre-processing steps completed by GEE include border noise removal, thermal noise removal, radiometric calibration, and terrain correction (Gorelick et al., 2017).

Synthetic Aperture Radar (SAR) interferometric imagery is useful in identifying wetlands, flooded areas, and separating water and land boundaries (Gulácsi and Kovács, 2020; Mohammadimanesh et al., 2018; Valenti et al., 2020). SAR data avoids issues with cloud and aerosol contamination common to other remote sensing data but is associated with higher processing requirements (Gulácsi and Kovács, 2020; Muro et al., 2019). The European Space Agency (ESA) launched the Sentinel-1 SAR interferometric paired satellites in April 2014, which have an average return frequency of 12 days globally and a 10-m band resolution (Torres et al., 2012). Timeseries of Sentinel-1 imagery have been shown to effectively monitor and delineate wetlands and standing water from surrounding upland and non-flooded areas (Hu et al., 2020; Muro et al., 2016; Xing et al., 2018). Sentinel-1 images from January 1, 2016, through December 31, 2021 were composited and 10th, 50th, and 90th percentiles of those stacks for the VV, VH, and band ratio (VV/VH) were calculated in GEE (band percentiles referred to by _p10, _p50, and _p90, respectively). A summary of the number of images per HUC-2 can be found in Table 1. The 6-year composite and percentile values were chosen to avoid disturbances from speckle noise (Bruggisser et al., 2021; Yang et al., 2021). The 10th and 90th percentile of the SAR bands have been shown to effectively identify permanent surface waters, while avoiding noise from speckle (Elkrachy et al., 2021; Kacic et al., 2021).

### 2.5. Model setup

The RF algorithm was chosen in this study for its flexibility to input data types, the algorithm’s stability given data noise, and avoidance of overfitting (Hoekstra et al., 2020; Mahdianpari et al., 2017b; Teluguntla et al., 2018). Random forest models have been shown to outperform other ML models as applied to large-scale studies and studies looking specifically at identifying wetlands (Berhane et al., 2018; Oliveira et al., 2012; Teluguntla et al., 2018). Predictor variables were selected to support the research goal of identifying PWA by delineating landscape features that support wetland habitat, rather than only identifying current wetland presence (Table 3). For example, the Normalized Difference Vegetation Index (NDVI) is frequently used in identifying existing wetlands and was intentionally not included as a predictor variable for this study in order to maximize the model identifying areas suitable to wetland habitat that may not be a current wetland. The wetlands binary raster, along with PWS, VOFD, HOFD, POFD, and CTI, were imported into GEE. VOFD, HOFD, POFD, and CTI were split into 44 55,808-pixel square tiles across CONUS, to meet the 10GB maximum upload size into GEE. These tiles were uploaded as needed, per HUC-2 model run.

The stratified sample method in GEE was used to create random training points within each HUC-2 with equal sampling between wetland and non-wetland classes based on the wetlands binary raster (Gorelick et al., 2017). The number of points were proportional to the total area of the HUC-2, with approximately 1 point per 475 km^2^, a rate comparable to other large-scale studies utilizing RF (Gumma et al., 2020; Mahdianpari et al., 2020a; Millard and Richardson, 2015; Teluguntla et al., 2018) (Table 1). Balanced training data has been shown to yield lower error rates and higher accuracy rates for minority classes, particularly in cases where the data are highly imbalanced, as is the case of wetland to non-wetland within HUC-2 watersheds (Mellor et al., 2015; Shetty et al., 2021). Using balanced training data in the case of imbalanced classes, as in this study, reduced bias towards the majority non-wetland class (Millard and Richardson, 2015). Further, because this study seeks to identify potential wetlands, balanced training data was used to consider the unknown proportion of potential wetlands across CONUS.

Two parameters were tested and set in the random forest classifier in GEE – the number of trees and the number of variables per node split. The default for the number of variables per split was used, which is the square root of input variables (17). This setting is largely supported in the literature (Pelletier et al., 2016; Shetty et al., 2021). The number of trees were set to 200, which has been shown to be sufficient in countrywide studies (Mahdianpari et al., 2017b).

### 2.6. Accuracy assessment

The training points for each HUC-2 were randomly divided into two sets by generating a random number column assigning each point a value between 0 and 1. The training set is then separated into two groups - approximately 70 % of the points (i.e., the random column value is less than 0.7) were used to train the RF models (training set) and the remaining approximately 30 % of points were retained for validation and were excluded from training our models (validation set).

To assess the accuracy of our modeled PWA, we trained an RF model using only the training set and applied this model to the reserved validation set. A confusion matrix identifying correctly and incorrectly labeled pixels in both the wetland and non-wetland class was generated from the values assigned to the reserved validation set. The values from the confusion matrix were used to calculate rates of commission errors, omission errors, and overall accuracy. The model to develop our PWA spatial data used pixel values from the wetlands binary raster and 17 predictor variables that intersected the training points (i.e., 100 % of the stratified sample points) to train a final RF model per HUC-2. The selection of training and validation points, applying the RF algorithm, and completing accuracy assessment in GEE was based on code from Qu et al. (2021).

The accuracy assessment compares our modeled results to our training dataset, which is based on the NWI. Because our input variables were selected to identify locations on the landscape with biophysical characteristics that support wetland habitat, the accuracy relative to the NWI is an indicator that our model is finding locations that support current wetlands based on these selected landscape characteristics. The PWA that does not intersect with the NWI represents our model’s predictive power, highlighting areas with topography, soils, and hydrology like existing wetlands, but not currently identified as wetland by the NWI.

### 2.7. Post-processing

**Fig. 1.**
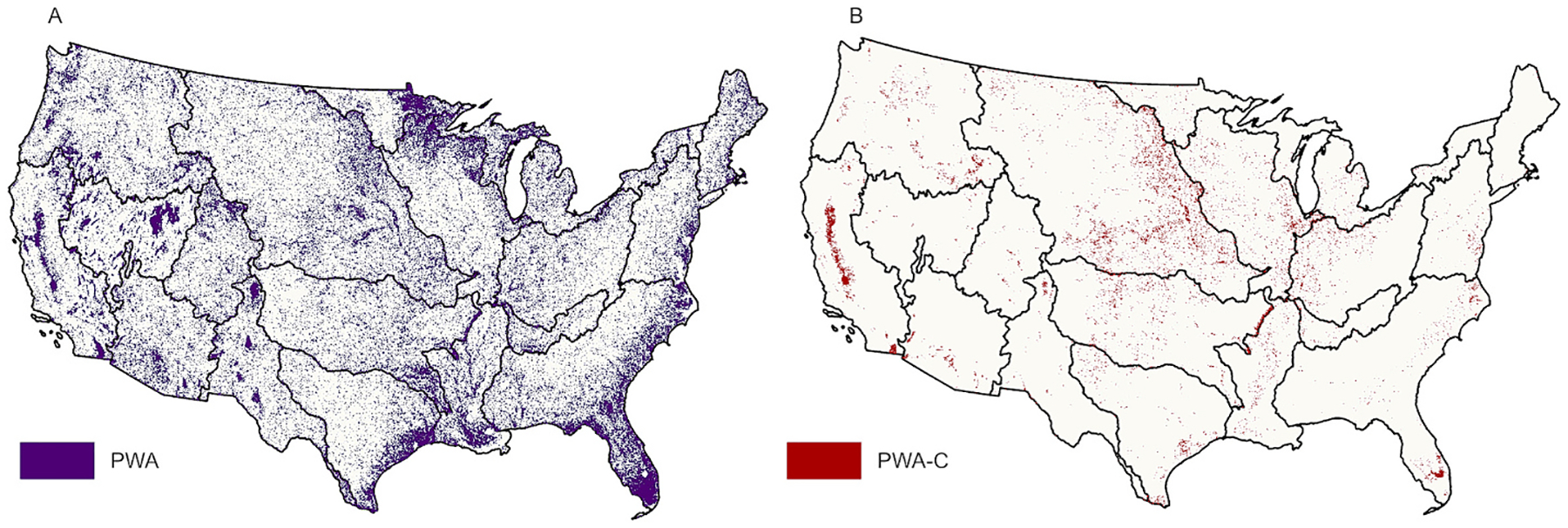
a. Model output for CONUS potential wetland area (PWA), 1b. Model output for CONUS PWA, intersected with Cultivated Crops to yield PWA–C. (2-column fitting image).

RF models for each HUC-2 were processed in GEE and the resulting spatial classification was exported in tiles. Post-processing was completed in ArcGIS Pro 2.8. The RF classifications were mosaicked by HUC-2 and projected to Albers Conical Equal Area to produce a CONUS 10-m PWA layer (Fig. 1a). The CONUS product was also assessed using the Region Group tool in ArcGIS Pro using the 8-neighbor method, which looks for connection via any side or vertex of a pixel. This yields a layer identifying wetland pixel clusters with the number of pixels in each cluster (Table 6).

The CONUS PWA product was overlaid on the Cultivated Crop land class from the 2019 National Land Cover Dataset (NLCD). The NLCD provides a 30-m LULC raster for CONUS, produced from Landsat imagery (Dewitz and U.S. Geological Survey, 2021). This produced a CONUS 10-m PWA layer on cropland (PWA–C, Fig. 1b).

## 3. Results

### 3.1. Model performance

The model predicts 1,663,808 km^2^ of PWA, which represents 21.1 % of CONUS land (Table 4). Of this, 67.9 % of the predicted potential wetland pixels do not intersect NWI wetland features. This segment of the modeled PWA, which accounts for 14.3 % of CONUS land cover, is the most valuable to this project as it identifies areas that are not currently identified as wetlands by the NWI but have considerable landscape-scale characteristics that indicate suitability for a wetland ecosystem.

The average overall accuracy of the validation sets for CONUS is 83.6 %, and HUC-2 overall accuracies range from 78.0 % to 89.8 % (Table 5). Of the NWI, 72.8 % intersects the modeled PWA, which accounts for 533,659 km^2^, or 6.8 % of CONUS land cover. From the training data extracted from NWI, the wetland class was identified as wetland by the model in 84.1 % cases, the non-wetland class was identified as wetland in 19.3 % cases, and the wetlands that were not included in training were identified as wetland in 87.7 % cases. These results show that the selected NWI wetland subsystems are identified correctly at a high rate by the model and those classes that were not considered wetland in the training dataset are also identified by the model.

While our validation set was used to assess accuracy, we also calculated the same accuracy statistics on our training set for three HUC-2’s that represent a diversity in watershed size and characteristics (New England Region, South Atlantic-Gulf Region, Great Basin Region) to ensure our models were appropriately tuned. Overall accuracy on the training sets for those three HUC-2 s (88.5 %, 88.2 %, and 90.5 %, respectively) were consistent with the accuracy of the validation sets (Table 5).

The Souris-Red-Rainy Region is a HUC-2 in which the wetland class commission error was lowest compared to other HUC-2 s, while the omission error was highest. This means that the modeled PWA show high intersection with the NWI training data wetlands, but that NWI wetlands are more prevalent where the model placed non-wetland pixels.

The average size of all potential wetland area pixel clusters produced by the RF algorithm is 126.3 pixels, or 0.01 km^2^, with a standard deviation of 78 km^2^ (Table 6). This average and standard deviation are more than double the same values for NWI pixel clusters, which have an average size of 0.005 km^2^ and standard deviation of 32 km^2^. The PWA pixels that do not intersect with NWI features are smaller, for both average and maximum pixel clusters, and the data are skewed towards smaller pixel cluster sizes. This indicates that our model places PWA around edges of NWI wetlands, that then provide for connection with nearby PWA pixel clusters.

### 3.2. Variable importance

**Fig. 2.**
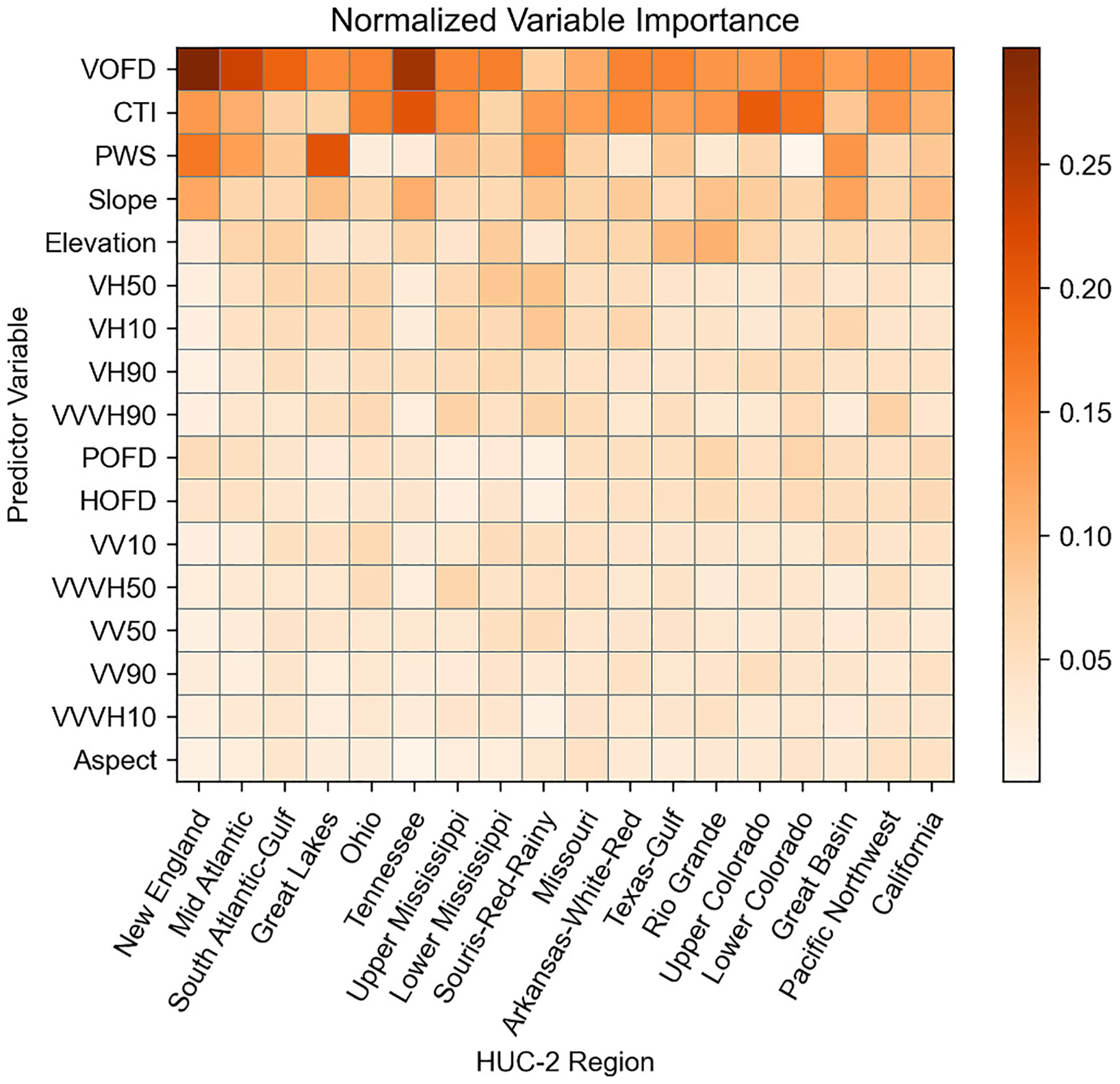
Variable importance by HUC-2, where darker colors indicate a higher normalized variable importance within the HUC-2. (1.5 column fitting image).

The normalized variable importance was calculated by scaling each variable’s importance to the overall model predictions against the remaining variables for each HUC-2 to total 1 (i.e., explains 100 % of the model’s results, Fig. 2). VOFD was the most important variable in 61 % of HUC-2 s (11) and second most important in an additional 33 % of HUC-2 s (6). CTI appeared within the top five most important variables in every HUC-2 across CONUS. Aspect performed worst across the study area, performing no better than 11th most important. The Sentinel-1 SAR bands performed similarly across CONUS. The VH median band was the top performing band in six HUC-2 s, with an average rank of 8.3, followed by the VH 10th and 90th percentile, with average ranks of 8.8 and 9.4, respectively. Slope was the most important elevation variable, with an average rank of 4.3. PWS performed as the top variable in three HUC-2 s, and the least important variable in the Lower Colorado Region HUC-2.

### 3.3. PWA on cropland

The 12.3 % of PWA that intersected NLCD’s Cultivated Crops class was extracted as PWA–C. PWA-C accounts for approximately 204,471 km^2^ of 1,318,235 km^2^ cropland, or 2.6 % of CONUS land area (Fig. 1b, Table 4). The Missouri Region HUC-2 has the most PWA-C coverage, at 5.4 % of the HUC-2 area. The New England Region has the smallest HUC-2 cropland coverage and the smallest PWA-C coverage at less than 0.2 %. The relationship between cropland and PWA-C coverage is not linear, illustrated by the two HUC-2 s with the most cropland coverage, which are ranked third and ninth in PWA-C coverage.

## 4. Discussion

The models used to identify 21.1 % of CONUS as PWA have an 83.6 % overall accuracy. The improvements in methodology and accuracy, as well as resolution at the CONUS-scale, will help policymakers and landowners to site wetland construction and restoration projects. This study seeks to update a previous project by Horvath et al. (2017). This prior work found 16.6 % of CONUS to have PWA area, and of that only 7.2 % being “high potential”. The previous study assessed overlap with NWI in 11 states as representative of accuracy, which ranged from 39 % to 95 %, including low, moderate, and high indicators of PWA.

The previous iteration and the current study both attempt to identify PWA across CONUS, but there are notable differences in methodology and accuracy assessment. The scale change from 30-m to 10-m increases the number of total cells in CONUS from 8.6 × 10^9^ to 7.9 × 10^10^. Additionally, the previous study utilized thresholds for CTI and the percentage of poorly drained or very poorly drained soils that were generated utilizing NWI wetlands from only two states, while this study utilized the RF algorithm to select PWA, and new training points were selected for each HUC-2. The previous iteration used NLCD’s Cultivated Crops and Hay / Pasture classes to identify PWA–C, and found 6.3 % of CONUS to be PWA–C, with 2.5 % of CONUS as “high potential” PWA–C. The current study did not use the Hay/Pasture NLCD class, as the class definition is broad enough to include different types of land ownership, which may not be accessible for government incentives. In the more constrained Cultivated Crops-only intersection with PWA, 2.6 % of CONUS is PWA–C. The high accuracy values in this study indicate that the modeled outputs correctly identified NWI wetlands across the entire study area, compared to the prior study which only looked at NWI overlap in 11 states.

Our study used methods to identify places on the landscape that have the biophysical characteristics likely to support wetland habitat, including existing wetlands, by our choices of input variables and training data. Others have used a similar approach to map existing wetlands for each of 15 ecozones encompassing Canada, for example (Mahdianpari et al., 2021). These researchers mapped existing wetlands using GEE and RF and used ground-truthing methods to measure their model accuracies in three iterations (Mahdianpari et al., 2021; Mahdianpari et al., 2020a; Mahdianpari et al., 2020b). Many others have used similar methods and variables to map existing wetlands at different scales, from individual delineated sites to watershed to basin (Babbar-Sebens et al., 2013; Berhane et al., 2018; O’Neil et al., 2018).

Other researchers have applied approaches analogous to our study using RF to model the biophysical characteristics associated with current ecosystems to identify areas with conditions suitable to support those same ecosystems in the future. Hughes et al. (2022) modeled the characteristics of present-day mangrove locations to predict where mangroves could migrate as a result of future sea level rise in New South Wales, Australia. Jiang et al. (2022) modeled existing tree cover across China using soil, climate, and topography variables to evaluate carbon stock restoration potential for areas without existing tree cover. Thus, our project combines methods and variables from studies that identify current wetlands, with methods and variables that can predict areas for wetland habitat based on landscape biophysical characteristics.

Further improvements for this work would be to include more variables that have been shown to support identifying wetland-supporting locations on the landscape. A project mapping the wetland restoration potential at a 3-m resolution across the state of Minnesota includes additional hydrologic position and topographic position indices, for example, which could improve model performance for a CONUS product (Johnson et al., 2024). Future work can also include additional accuracy testing against the statewide National Wetlands Inventory update in Minnesota, Johnson et al.’s (2024) Minnesota Restorable Wetland Index, and sources that have access to restored wetland sites (e.g., U.S. Army Corps of Engineers) (Kloiber et al., 2019). Improved existing wetlands data would be useful to further identify locations on the landscape that are not currently a wetland but exhibit biophysical characteristics of a wetland habitat.

There are limitations inherent with the data used to support our study. The quality of the NWI varies across the country, with some imagery from the 1970s, and other sections of the U.S. having not been mapped by the NWI at all. This lack of standardized, current wetlands data across CONUS limits the quality of training data. This also impacts our accuracy assessment, as some locations identified by our model to be PWA may be existing wetland that are not correctly identified in the NWI. Because machine learning relies on training data, improvements in these input data are critical to improving this work. The extent of the 3DEP digital elevation model limited the flow accumulation and direction calculations. This resulted in some NoData areas along the Canada and Mexico borders where flow accumulation could not be calculated, as flow direction was towards the CONUS boundaries, but no channel in that direction could be identified. These NoDatas made up 0.009 % of CONUS area. While all model input variables had a resolution of 10-m, the NLCD products are at 30-m, and finer resolution cropland data may have slightly changed the distribution of PWA–C.

Finally, as with any model, there are limitations in our model’s design and uses. While our accuracy measures are useful for evaluating how often the model correctly classifies areas in the training data, it is important to note that the actual accuracy of all PWA is not being measured. This is primarily because the true coverage of PWA on the ground is unknown. In addition to limiting the measurement of our accuracy assessment, not having information on the full extent of PWA could introduce bias into our RF sampling and modeling decisions. We chose to artificially use balanced class samples so that wetland and non-wetland samples were equally represented in each HUC-2 model. Previous research has found that RF outputs will predict more of a given class if a higher proportion of training data than exists on the ground is included (Mellor et al., 2015; Millard and Richardson, 2015). Similarly, we relied on values from previous studies to set our RF hyperparameters. Further research on actual PWA on the ground relative to the study area could better inform these decisions, particularly to better represent regional differences in each HUC-2. Our PWA product includes existing wetlands, both those that are mapped in the NWI and those that are not mapped in the NWI, along with areas suitable for restoration or construction of wetlands. Users may overlay the outputs of this study with NWI or improved wetland location data to identify areas best suited for restoration or construction of wetlands.

## 5. Conclusion

Wetland ecosystems are valuable assets that have historically been drained and filled across the United States. This study utilized machine learning and big data to identify areas that are likely able to support built or restored wetland habitats at a finer resolution than previously available for CONUS. Utilizing 10-m data not only vastly increases the number of cells evaluated by the model, but also allows finer details on the landscape to become visible that are not seen at the 30-m scale. This can assist programs like WREP to better inform participants on wetland placement in their specific landscape conditions. Cheng et al. (2020), for example, found that with a 10 % increase in wetlands placed where nitrogen surplus was the highest, nitrogen removal increased 90 % over current levels. The PWA product may be used by land planners to target areas that would both benefit from a wetland ecosystem (e.g., areas with excess nitrogen) in terms of need for a particular ecosystem service and areas that would yield successful wetland habitats. Further analysis on the clustering of PWA wetlands can highlight areas that can support larger connected systems, particularly with respect to providing ecosystem services for areas more prone to degradation. While we intersected PWA with a cropland dataset, this product can be overlaid with other land use types, scenarios, or features to view the best sites for wetland area.

The RF models show 1,663,808 km^2^, or 21.1 % of CONUS as PWA, a significant increase from the 16.6 % PWA seen in previous estimates. Of the PWA, 204,471 km^2^ were identified as PWA–C, accounting for 15.5 % of croplands, and 2.6 % of CONUS area. The performance of RF models across CONUS yielded an 83.6 % overall accuracy of identifying existing NWI wetlands and included significant areas outside of the NWI as PWA. Future work could follow the success of restored or built wetlands, and their associated auxiliary landscape characteristics, to better inform wetland potential modeling. Ground-truthing the model results may also help locate areas that the model identified as PWA that are an existing wetland. The products from this study are an invaluable tool for directing and refining the process for siting wetland construction or restoration.

## Supplementary Material

Supplement1

## Figures and Tables

**Table 1:** Characteristics of each HUC -2 in CONUS and associated modeling components (Dewitz And U.S. Geological Survey, 2021; United States Fish And Wildlife Service, 2021).

HUC-2 name	Area (km^2^)	Total Training points (N)	Total Validation set points^a^ (N)	Total Sentinel-1 images (N)	NWI area (%)	Cultivated Crops area (%)^b^
New England region	163,110	685	199	2857	17.2	0.9
Mid Atlantic region	276,189	1160	325	3521	15.9	8.1
South Atlantic-Gulf region	724,336	3042	862	6644	23.4	8.2
Great Lakes region	325,421	1367	398	6038	17.9	23.3
Ohio region	421,962	1772	539	4480	3.8	22.2
Tennessee region	105,950	445	148	1987	5.7	4.3
Upper Mississippi region	492,027	2067	620	5526	11.7	49.4
Lower Mississippi region	276,482	1161	332	3808	27.1	26.9
Souris-Red-Rainy region	155,063	651	220	3355	23.0	49.9
Missouri region	1,322,148	5553	1673	10,647	4.2	27.5
Arkansas-White-Red region	642,284	2698	801	5798	4.1	18.9
Texas-Gulf region	471,091	1979	632	4602	6.7	13.3
Rio Grande region	343,229	1442	445	4366	1.7	1.3
Upper Colorado region	293,569	1233	372	3036	2.7	1.2
Lower Colorado region	362,982	1525	452	3204	2.1	1.6
Great Basin region	367,049	1542	460	4314	8.7	1.9
Pacific Northwest region	723,646	3039	912	6316	5.5	8.0
California region	430,658	1809	534	6615	8.5	9.1
***CONUS***	***7,897,197***	***33,168***	***9924***	***55,509***	***9.3***	***16.3***

a Validation set points are a random selection of approximately 30 % of the total training points.

b Cultivated Croplands class from 2019 NLCD landcover Product (Dewitz And U.S. Geological Survey, 2021).

**Table 2:** Designation of National Wetlands Inventory subsystems used to create the training data (United States Fish and Wildlife Service, 2021).

Wetland	Riverine intermittent
	Lacustrine littoral
	Palustrine aquatic bed
	Palustrine farmed
	Palustrine forested
	Palustrine moss-lichen
	Palustrine rock bottom
	Palustrine scrub-shrub
	Palustrine unconsolidated bottom
	Lacustrine diked/impounded
	Lacustrine excavated
Non-wetland	Marine subtidal
	Marine intertidal
	Estuarine subtidal
	Estuarine intertidal
Not Included	Riverine tidal
	Riverine lower perennial
	Riverine upper perennial
	Riverine unknown
	Lacustrine limnetic
	Lacustrine

**Table 3:** Description, date, and supporting literature of data used for input and target variables.

Variable name	Short name	Variable description	Derivative source (date)	Variable type	Supporting citations
Predictor variables					
Synthetic Aperture Radar	VV10	VV-band 10th percentile	European Space Agency Sentinel −1 Mission (Torres et al., 2012) (1/2016–12/2021)	Continuous	Gulácsi and Kovács (2020); Mahdianpari et al. (2017a); Mohammadimanesh et al. (2018)
	VV50	VV-band 50th percentile			
	VV90	VV-band 90th percentile			
	VH10	VH-band 10th percentile			
	VH50	VH-band 50th percentile			
	VH90	VH-band 90th percentile			
	VVVH10	VV/VH ratio 10th percentile			
	VVVH50	VV/VH ratio 50th percentile			
	VVVH90	VV/VH ratio 90th percentile			
Topographic	Elevation	Elevation	USGS 3DEP (2021)	Continuous	Dubeau et al. (2017); Higginbottom et al. (2018);
	Slope	Slope			Uuemaa et al. (2018)
	Aspect CTI	Aspect Compound Topographic Index			
	HOFD	Horizontal overland flow distance			
	VOFD	Vertical overland flow distance			
	POFD	Pythagoras overland flow distance			
Soil	PWS	Potential Wetland Soils	Soil Survey Staff, Natural Resources Conservation Service, U.S. Department of Agriculture (2021a, b)	Percentage	Corcoran et al. (2013)
Target variable					
Wetland/Non-wetland	NWI	Binary wetlands raster	United States Fish and Wildlife Service (2021)	Categorical	Muñoz et al. (2019)

**Table 4:** Modeled potential wetland area (PWA) areal coverage and percent coverage by HUC-2 and CONUS.

HUC-2	PWA Within HUC-2 (km^2^)	% HUC-2 PWA Area	PWA-C Within HUC-2 (km^2^)	% HUC-2 PWA-C Area
New England Region	38,941	23.9	293	0.2
Mid Atlantic Region	49,191	17.8	3085	1.1
South Atlantic-Gulf Region	219,000	30.2	10,062	1.4
Great Lakes Region	87,219	26.8	4766	1.5
Ohio Region	71,966	17.1	11,595	2.7
Tennessee Region	19,733	18.6	1546	1.5
Upper Mississippi Region	111,433	22.6	20,108	4.1
Lower Mississippi Region	71,506	25.9	6651	2.4
Souris-Red-Rainy Region	40,151	25.9	2397	1.5
Missouri Region	252,945	19.1	71,144	5.4
Arkansas-White-Red Region	121,043	18.8	18,401	2.9
Texas-Gulf Region	90,988	19.3	8548	1.8
Rio Grande Region	68,869	20.1	3061	0.9
Upper Colorado Region	61,588	21.0	1892	0.6
Lower Colorado Region	73,612	20.3	4119	1.1
Great Basin Region	65,259	17.8	1690	0.5
Pacific Northwest Region	136,109	18.8	15,340	2.1
California Region	84,231	19.6	19,776	4.6
***CONUS***	1,663,803	21.1	204,472	2.6

**Table 5:** Rates of commission error (CE) and omission error (OE), and overall accuracy on the validation data by HUC and CONUS.

HUC-2	Non-wetland		Wetland		Overall accuracy
	CE	OE	CE	OE	
New England Region	17.4	10.9	12.2	19.4	84.9
Mid Atlantic Region	19.3	16.1	15.2	18.2	82.8
South Atlantic-Gulf Region	15.0	15.6	15.8	15.2	84.6
Great Lakes Region	14.0	15.3	14.8	13.5	85.6
Ohio Region	14.2	14.9	15.5	14.8	85.2
Tennessee Region	6.8	17.9	20.3	7.8	86.5
Upper Mississippi Region	13.4	13.4	12.5	12.5	87.1
Lower Mississippi Region	23.5	20.1	19.3	22.5	78.6
Souris-Red-Rainy Region	21.9	6.1	8.4	28.3	83.2
Missouri Region	15.5	15.0	15.2	15.7	84.6
Arkansas-White-Red Region	19.6	16.0	17.2	21.0	81.5
Texas-Gulf Region	16.1	13.7	14.6	17.0	84.7
Rio Grande Region	19.0	23.8	25.0	20.0	78.0
Upper Colorado Region	15.2	21.2	19.4	13.8	82.5
Lower Colorado Region	19.9	23.5	21.6	18.2	79.2
Great Basin Region	10.8	8.9	9.5	11.6	89.8
Pacific Northwest Region	14.9	16.2	16.9	15.6	84.1
California Region	22.6	18.6	19.9	24.2	78.7
***CONUS***	16.5	16.0	16.3	16.9	83.6

**Table 6:** Statistics about pixel clusters of wetlands in the National Wetlands Inventory (NWI), the modeled Potential Wetland Area (PWA), and those pixels that are PWA that do not overlap with NWI pixels.

	Minimum pixel cluster size (km^2^)	Maximum pixel cluster size (km^2^)	Mean pixel cluster size (km^2^)	Median pixel cluster size (km^2^)	5th Percentile pixel cluster size (km^2^)	95th Percentile pixel cluster size (km^2^)	Standard Deviation (km^2^)	Proportion of area made of 0.0001 km^2^ wetlands
NWI-only	0.0001	415,685	0.005	0.0002	0.0001	0.0005	32	0.5
PWA-only	0.0001	821,586	0.01	0.0002	0.0001	0.0007	78	0.2
PWA without NWI	0.0001	12,868	0.008	0.0002	0.0001	0.0004	3	0.5

## Data Availability

The products of this study are posted on the U.S. EPA’s EnviroAtlas, a public-facing repository of spatial data layers that relate to human health and ecosystem services (https://www.epa.gov/enviroatlas). The spatial data are also available for download (https://doi.org/10.23719/1531228).

## HIGHLIGHTS

- Landscape-scale biophysical characteristics can support wetland conservation goals.
- Access to high-resolution data and tools for analysis increases modeling capacity.
- Random forest algorithm was applied to 18 regional watersheds at 10-m resolution.
- Potential Wetland Area covers 21.1 % of conterminous United States land.
- Model overall accuracies range from 78.0 % to 89.8 %, with an average of 83.6 %.
