# Supplementary material for: Advancements in mapping areas suitable for wetland habitats across the conterminous United States
Source: Sci Total Environ. Author manuscript; Available in PMC 2025 Nov 1. (PMC11998124; doi:10.1016/j.scitotenv.2024.175058)
Supplement: Supplement1 [file NIHMS2069517-supplement-Supplement1.docx]

Advancements in mapping potential wetland areas across the Conterminous United States

Lauren Krohmer^a,c^, Elijah Heetderks^a,c^, Jeremy Baynes^b^, Anne Neale^a^

^a^ US Environmental Protection Agency (EPA), Center for Public Health and Environmental Assessment (CPHEA), Environmental Pathways Modeling Branch (EPMB), 109 T.W. Alexander Drive, Research Triangle Park, NC 27711, USA

^b^ US Environmental Protection Agency (EPA), Center for Public Health and Environmental Assessment (CPHEA), Environmental Pathways Modeling Branch (EPMB), 109 T.W. Alexander Drive, Research Triangle Park, NC 27711, USA. [Baynes.Jeremy@epa.gov](mailto:Baynes.Jeremy@epa.gov) (Corresponding author)

^c^ Oak Ridge Associated Universities, Oak Ridge, TN, USA

Supplementary Information


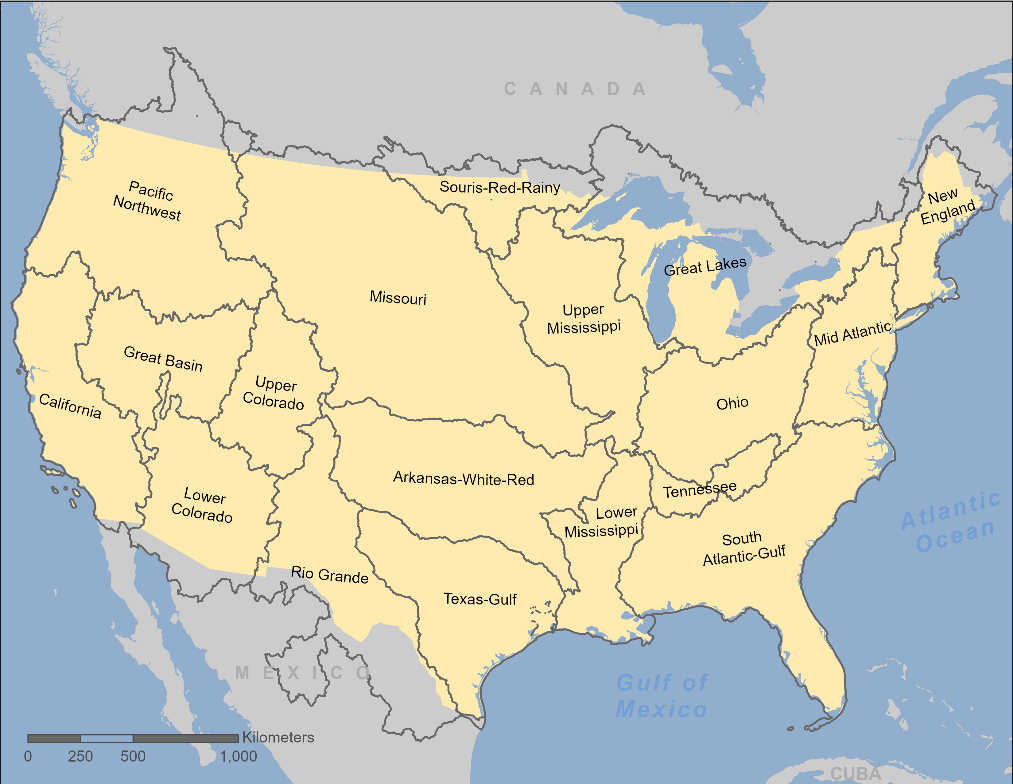


Fig. S1. Map illustrating the extent and name of the HUC-2 watersheds (black outline) and the extent of the model output (yellow) (U.S. Geological Survey 2013).


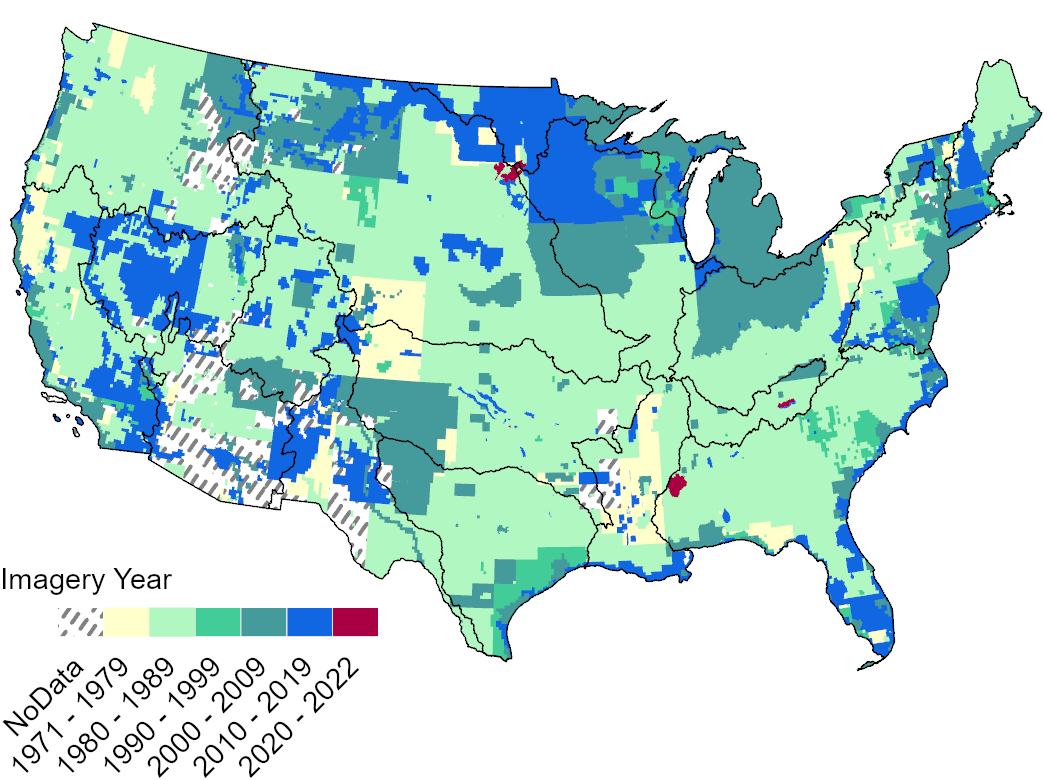


Fig. S2. Map illustrating the year that aerial imagery were sourced to identify wetlands in the NWI (United States Fish and Wildlife Service 2021).


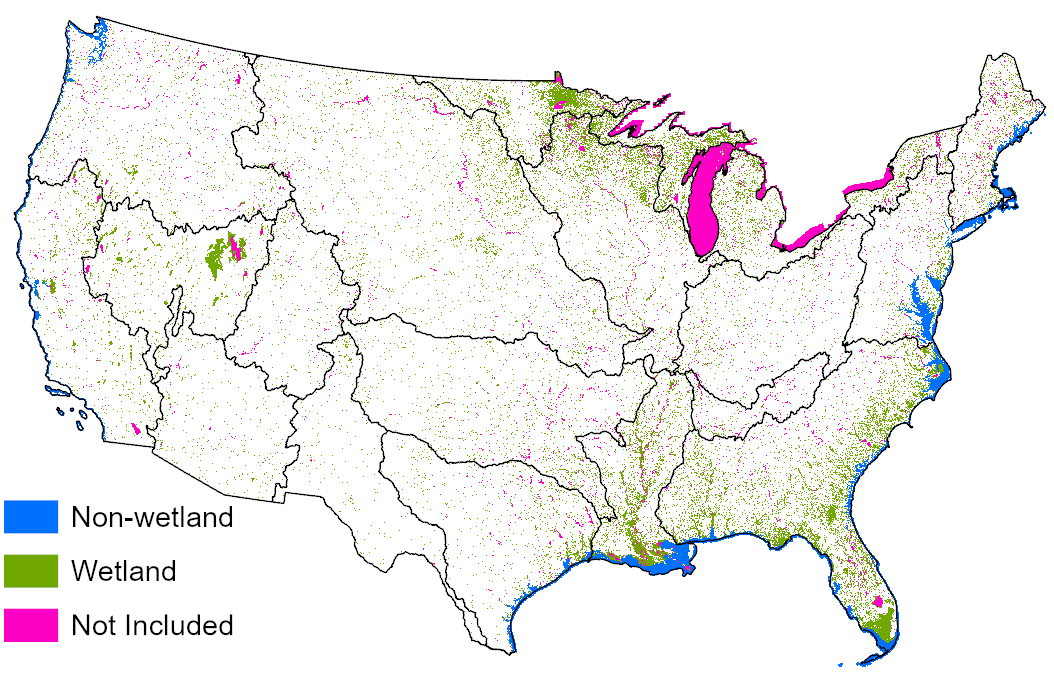


Fig. S3. Map illustrating the wetland subclasses from the National Wetlands Inventory (NWI) grouped by designation as wetland, non-wetland, or not included in the model training dataset (United States Fish and Wildlife Service 2021).


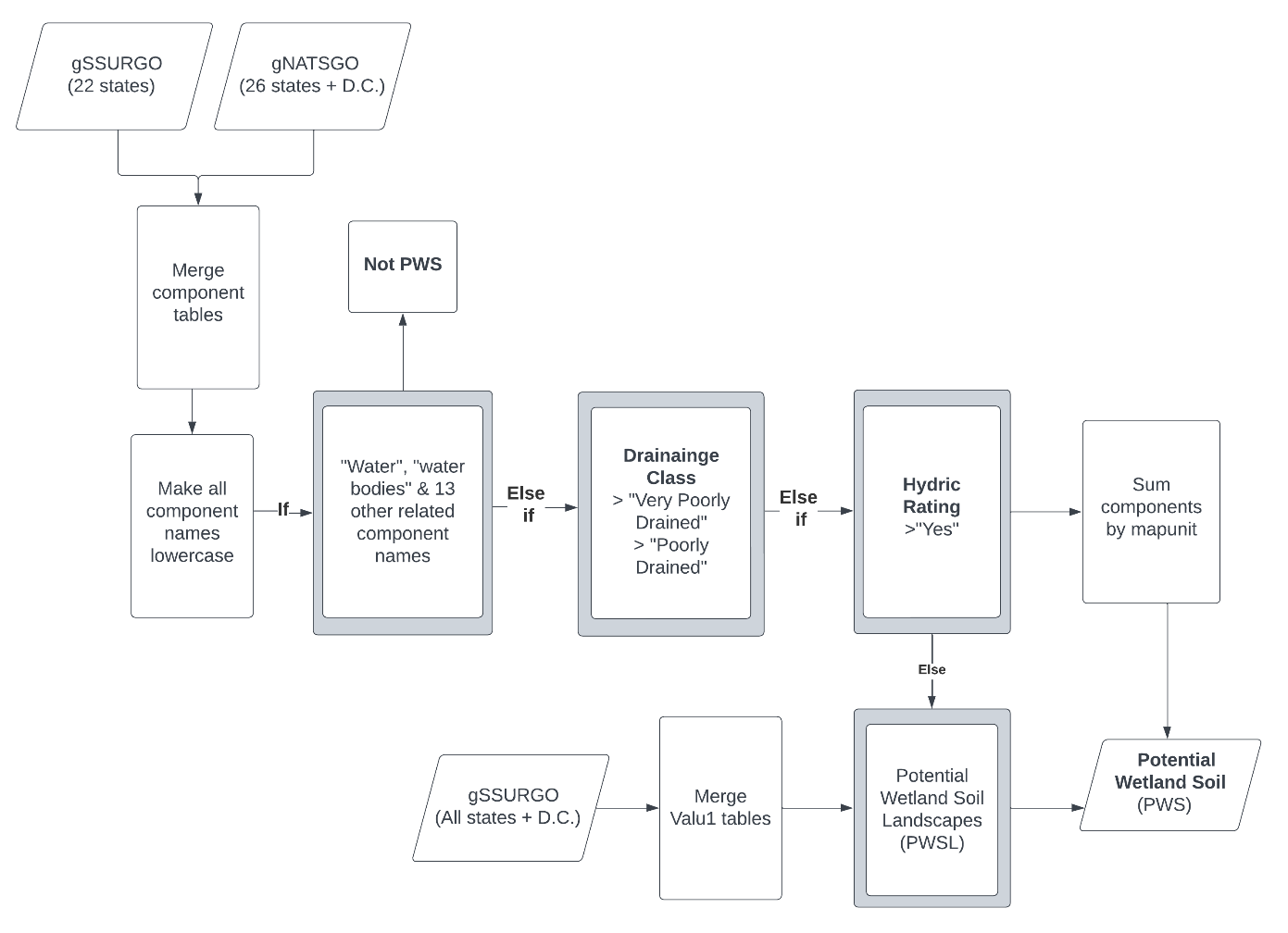


Fig. S4. Flow chart describing the methodology applied to the soil input variable (PWS). The chart outlines the prioritization of drainage class, hydric rating, and Potential Wetland Soil Landscapes (PWSL) used (Soil Survey Staff 2021a, 2021b).


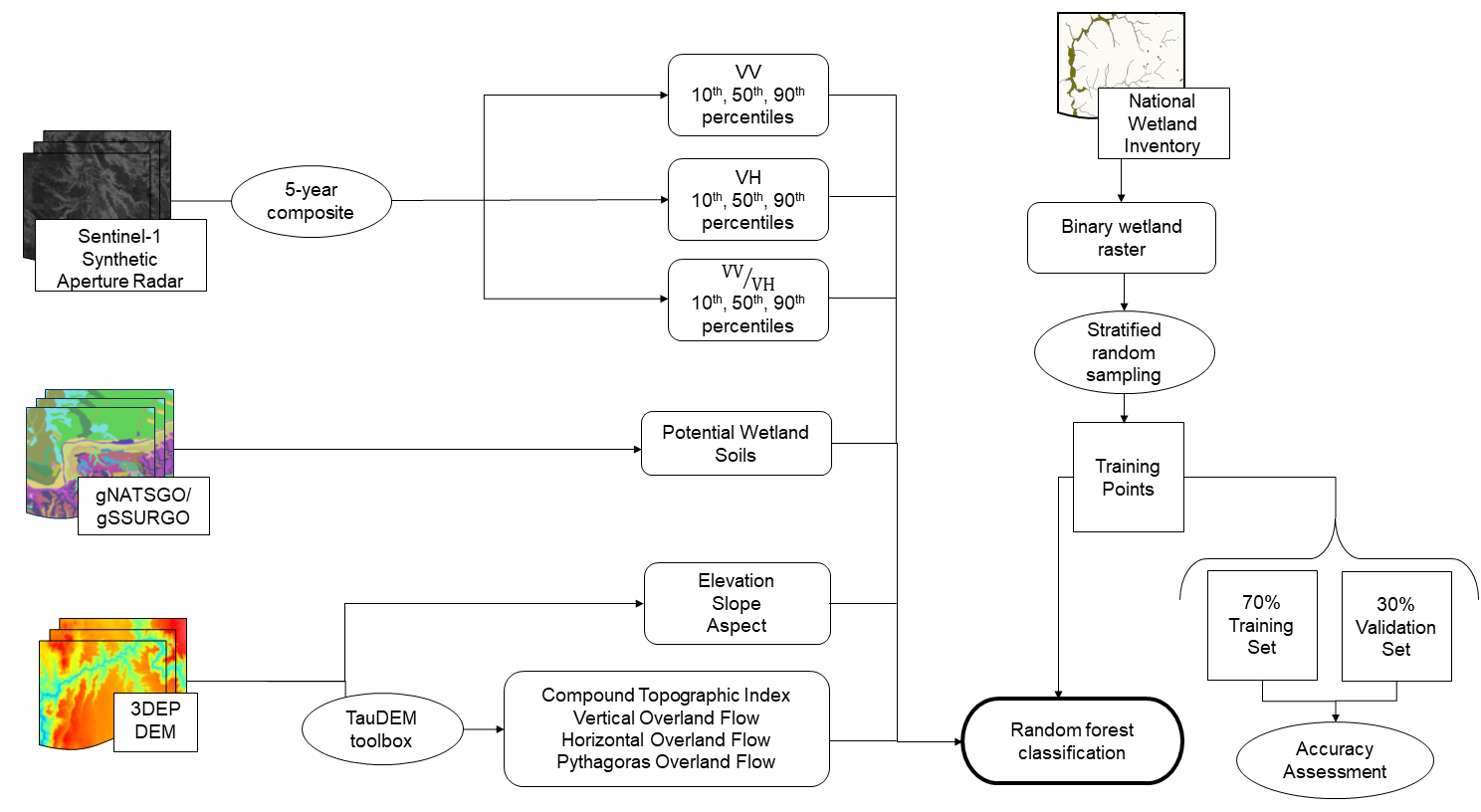


Fig. S5. Flow chart outlining the methods of this study, including input carriable development and random forest modeling.

| Supplementary Table 1 Accuracy assessment for the training set used to train random forest models for three HUC-2’s to ensure models are not overfitted. | | | | | |
| --- | --- | --- | --- | --- | --- |
|  | **Non-wetland** | | **Wetland** | |  |
| **HUC-2** | **CE** | **OE** | **CE** | **OE** | **Overall Accuracy** |
| New England Region | 10.8 | 12.3 | 12.2 | 10.6 | 88.5 |
| South Atlantic-Gulf Region | 10.6 | 13.9 | 13 | 9.8 | 88.2 |
| Great Basin Region | 11.2 | 7.4 | 7.7 | 11.7 | 90.5 |


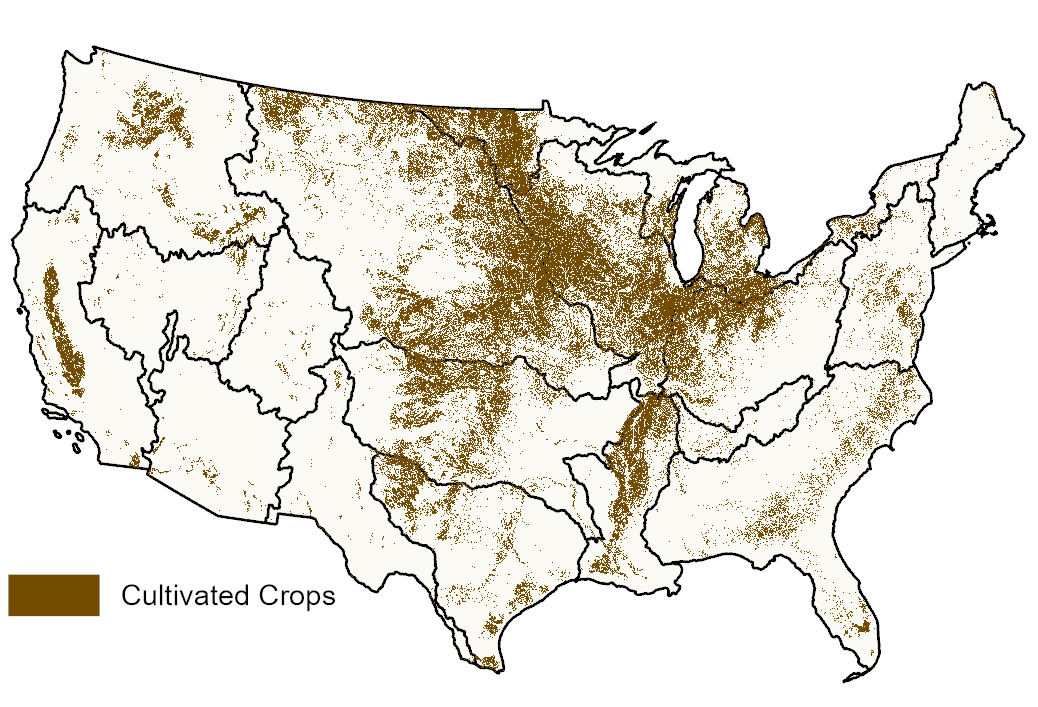


Fig. S6. 2019 NLCD Cultivated Crops class (Dewitz *et al.* 2021).

Dewitz, J. and U.S. Geological Survey (2021). National Land Cover Database (NLCD) 2019 Products (ver. 2.0, June 2021). U.S. Geological Survey data release.

Soil Survey Staff, N. R. C. S., United States of Agriculture (2021). Gridded National Soil Survey Geographic (gNATSGO) Database for AR,AZ,CA,CO,FL,GA,ID,KY,MI,MN,MT,NC,ND,NH,NM,NV,NY,OK,OR,TN,TX,UT,VA,VT,WA,WI, WY. Available online at <https://nrcs.app.box.com/v/soils>.

Soil Survey Staff, N. R. C. S., United States of Agriculture (2021). Soil Survey Geographic Database (SSURGO) for AL, CT, DC, DE, IA, IL, IN, KS, LA, MA, MD, ME, MO, MS, NE, NJ, OH, PA, RI, SC, SD, WV. Available online at <https://nrcs.app.box.com/v/soils>.

U.S. Geological Survey, U. S. D. o. A. N. R. C. S. (2013). Federal Standards and Procedures for the National Watershed Boundary Dataset (WBD). Techniques and Methods.

United States Fish and Wildlife Service. (2021). "National Wetlands Inventory." from <https://www.fws.gov/wetlands/>.
